# MALT1 protease activity in primary effusion lymphoma

**DOI:** 10.18632/oncotarget.23693

**Published:** 2017-12-26

**Authors:** Mélanie Juilland, Luca Bonsignore, Margot Thome

**Affiliations:** University of Lausanne, Department of Biochemistry, Epalinges, Switzerland

**Keywords:** paracaspase, NF-kB, PEL, herpes virus, kaposi

**Figure 1:**
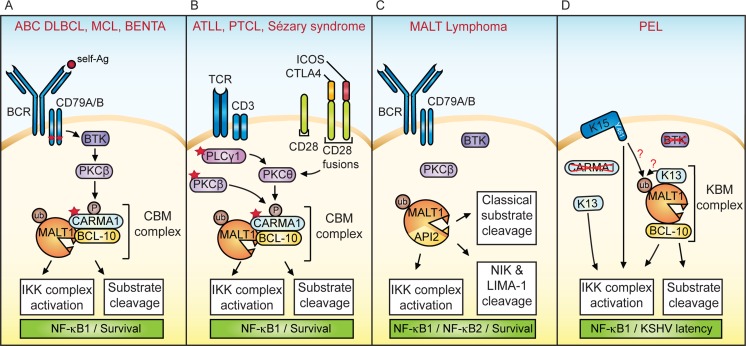
Constitutive MALT1 activation in lymphomas and lymphoproliferative disease

The activation, proliferation and survival of lymphocytes depend on the antigen receptor-induced formation of cytoplasmic signaling complexes that control specific transcriptional pathways. The protease MALT1 has an essential role in lymphocyte activation and lymphoma development [1, 2]. Upon antigen receptor triggering, MALT1 is recruited to the so-called CBM complex, formed by the assembly of the proteins CARMA1 (also known as CARD11), BCL-10 and MALT1. The CBM complex promotes the activation of the transcription factor NF-κB1 (also known as the classical NF-κB pathway) by two means: first, it allows MALT1 to recruit and activate the IκB kinase (IKK)-complex, which phosphorylates and thereby inactivates the NF-κB inhibitor IκB, and second, it promotes the MALT1-dependent cleavage of proteins with negative regulatory roles in the NF-κB1 pathway, namely A20 and RelB (Figure 1A and 1B). MALT1 inhibitors already showed promise in preclinical lymphoma studies [3] and recent findings from our laboratory now suggest they may also be efficient against virally induced lymphomas [4].

Over the last few years, constitutive MALT1 activation has been recognized as a common feature of various B cell malignancies and B cell proliferative diseases, such as diffuse large B-cell lymphoma of the activated B-cell subtype (ABC DLBCL), mantle cell lymphoma (MCL), MALT lymphoma and BENTA syndrome (Figure 1A and 1C) [2]. Recently, the proto-oncogenic role of MALT1 has been extended to several T cell malignancies, such as adult T-cell leukemia/lymphoma (ATLL), peripheral T cell lymphoma (PTCL) and Sézary syndrome (Figure 1B) [2, 5]. The addiction of these lymphomas to MALT1’s protease and scaffold activity is mainly due to oncogenic somatic mutations that alter the activity or expression of components of the CBM complex itself or of its upstream signaling events, and/or to the recognition of self-antigens [2], as depicted in Figure 1 (red stars indicate presence of mutations). A recurrent mutation found in MALT lymphoma is a chromosomal translocation that generates a MALT1-API2 fusion protein (Figure 1C). This fusion protein constitutively activates NF-κB1 through MALT1’s scaffold function and cleavage of classical MALT1 substrates. The API2 moiety of the MALT1-API2 fusion protein additionally recruits the kinase NIK and the tumor suppressor LIMA-1, whose cleavage promotes activation of the NF-κB2 pathway and cell growth, respectively [1, 2].

Lymphoid tumors can also be triggered by oncogenic herpes viruses, such as Epstein-Barr virus (EBV, also known as HHV-4), associated with Hodgkin and Burkitt lymphomas, or Kaposi’s sarcoma herpes virus (KSHV, also known as HHV-8), which induces primary effusion lymphoma (PEL) and multicentric Castleman disease-associated plasmablastic lymphomas [6]. Recently, we have proposed a possible role for MALT1 in the development of PEL (Figure 1D) [4]. PEL is a rare and incurable B-cell malignancy observed in immunocompromised patients. Malignant growth of KSHV-infected B cells requires the maintenance of viral latency and suppression of the viral lytic program. Amongst the KSHV latent genes, K13 and K15 are key regulators of NF-κB signaling [6]. K13 encodes a cytoplasmic protein of the FLIP family involved in inhibition of apoptosis and activation of NF-κB, while K15 encodes a membrane protein homologous to LMP2A (latent membrane protein 2A), an NF-κB inducing gene essential for EBV latency. K13 activates NF-κB1 through a physical interaction with NEMO/IKKγ that promotes the activation of the IKK complex (Figure 1D) [6]. K15, on the other hand, recruits and activates the Ser/Thr kinase NIK together with IKKα and β to activate NF-κB1. This requires an intact SH2-binding motif (Y481EEVL) in the cytoplasmic domain of K15 (Figure 1D) [6].

A recent study from our group has provided further insight into how K13 and K15 promote NF-κB1 activation and viral latency, and identified the protease MALT1 as a key regulator of KSHV latency and growth of PEL [4]. We found that treatment with Thioridazine, an allosteric MALT1 inhibitor [7], strongly affected cell viability of several PEL cell lines *in vitro* [4]. Additionally, MALT1 inhibitors led to a dramatic reduction in PEL development *in vivo* using a xenograft model. Amongst the 86 open reading frames (ORFs) of KSHV, we identified K13 and K15 as potent MALT1 activators [4]. K13 directly interacted with the protease domain of MALT1 and may thus induce an active (or activatable) MALT1 conformation. K15 most likely activated MALT1 indirectly, via the recruitment of (an) SH2-containing protein(s) to its cytoplasmic tail. Interestingly, the PEL cell lines used for this study lacked significant surface BCR expression and did not express the BCR signaling components BTK and CARMA1 [4]. PEL cells may thus use a KBM complex composed of K13, BCL-10 and MALT1, to activate MALT1 in a CARMA1-independent manner (Figure 1D). K15 might also contribute to KBM activation, and/or promote MALT1 activation through recruitment of unknown MALT1 activators [4].

In contrast to other lymphoid malignancies, where somatic mutations in cellular proteins activate MALT1 in a constitutive manner, KSHV thus promotes MALT1 activation by specific viral proteins that short-cut the requirement for CBM assembly. KSHV thereby exploits MALT1-dependent signal transduction to maintain its latency and escape the immune system. Consequently, MALT1 inhibition might be considered as an option to treat PEL patients, ideally in combination with an anti-herpes virus drug that prevents viral replication and spreading, such as ganciclovir. Of note, MALT1 may also play a role in the latency maintenance of other viruses. Indeed, inhibition of MALT1 induced massive cell death in latently HIV-infected T cells under conditions of concurrent cellular activation [8]. Inhibiting MALT1 activity might thus provide a more general therapeutic option to purge viruses such as KSHV and HIV from their latent reservoir.
